# Sperm DNA methylation patterns at discrete CpGs and genes involved in embryonic development are related to bull fertility

**DOI:** 10.1186/s12864-022-08614-5

**Published:** 2022-05-18

**Authors:** Miriama Štiavnická, Aurélie Chaulot-Talmon, Jean-Philippe Perrier, Petr Hošek, David A. Kenny, Patrick Lonergan, Hélène Kiefer, Sean Fair

**Affiliations:** 1 Department of Biological Sciences, Laboratory of Animal Reproduction, Biomaterials Research Cluster, Bernal Institute, Faculty of Science and Engineering, University of Limerick, Limerick, Ireland; 2 Université Paris-Saclay, UVSQ, INRAE, BREED, Jouy-en-Josas, France; 3 Ecole Nationale Vétérinaire d’Alfort, BREED, Maisons-Alfort, France; 4 Biomedical Center, Faculty of Medicine in Pilsen, Charles University, Pilsen, Czech Republic; 5 Animal and Bioscience Research Department, Animal and Grassland Research and Innovation Centre, Teagasc Meath, Ireland; 6 School of Agriculture and Food Science, University College Dublin, Belfield, Dublin 4, Ireland

**Keywords:** Male fertility, Dairy industry, DNA methylation, Epigenetics, RRBS, Spermatozoa

## Abstract

**Background:**

Despite a multifactorial approach being taken for the evaluation of bull semen quality in many animal breeding centres worldwide, reliable prediction of bull fertility is still a challenge. Recently, attention has turned to molecular mechanisms, which could uncover potential biomarkers of fertility. One of these mechanisms is DNA methylation, which together with other epigenetic mechanisms is essential for the fertilising sperm to drive normal embryo development and establish a viable pregnancy. In this study, we hypothesised that bull sperm DNA methylation patterns are related to bull fertility. We therefore investigated DNA methylation patterns from bulls used in artificial insemination with contrasting fertility scores.

**Results:**

The DNA methylation patterns were obtained by reduced representative bisulphite sequencing from 10 high-fertility bulls and 10 low-fertility bulls, having average fertility scores of − 6.6 and **+** 6.5%, respectively (mean of the population was zero). Hierarchical clustering analysis did not distinguish bulls based on fertility but did highlight individual differences. Despite this, using stringent criteria (DNA methylation difference ≥ 35% and a q-value < 0.001), we identified 661 differently methylated cytosines (DMCs). DMCs were preferentially located in intergenic regions, introns, gene downstream regions, repetitive elements, open sea, shores and shelves of CpG islands. We also identified 10 differently methylated regions, covered by 7 unique genes (*SFRP1, STXBP4, BCR, PSMG4, ARSG, ATP11A, RXRA*), which are involved in spermatogenesis and early embryonic development.

**Conclusion:**

This study demonstrated that at specific CpG sites, sperm DNA methylation status is related to bull fertility, and identified seven differently methylated genes in sperm of subfertile bulls that may lead to altered gene expression and potentially influence embryo development.

**Supplementary Information:**

The online version contains supplementary material available at 10.1186/s12864-022-08614-5.

## Background

Artificial insemination (AI) using the semen of elite sires is core to the genetic progress achieved in the dairy industry over the last number of decades. One of the long-standing challenges for the AI industry is the variation in bull field fertility where individual bulls can vary by over 20% points in pregnancy rate despite their semen passing stringent quality control checks prior to release into the field [1]. These checks are mainly based on the assessment of post-thaw sperm motility and morphology using subjective methods or more objective methods such as computer-assisted sperm analysis (CASA) [2, 3]. There is added value to the evaluation of bull semen by flow cytometric techniques that assess various sperm functional parameters such as membrane and acrosome integrity, oxidative stress and DNA fragmentation [4, 5]. However, reliable prediction of bull fertility based on in vitro sperm quality functional parameters is still not possible. The challenge has increased in recent years with the advent of genomics, as young bulls, with unproven field fertility, are now extensively used in their first season [6]. Although oocyte fertilisation rates following insemination in cattle are quite high (> 85%), pregnancy failures during early embryo development and implantation still occur [7–9]. While much of this embryo loss is likely attributable to issues associated with the female (oocyte quality, uterine receptivity), the sire also makes a significant contribution [10, 11].

More recently, there has been an increased focus on ‘Omics’-based techniques that could uncover hitherto unidentified causes of bull infertility [12–15]. It is well established that sperm are transcriptionally and translationally inactive with limited mechanisms for the regulation of gene expression [16]. Despite that, sperm can alter early embryo development through DNA methylation [17, 18], posttranslational modifications of histones [19–21] and non-coding RNAs [22, 23], which modify gene expression without actual changes in the DNA sequence.

The DNA methylation pattern of mature, ejaculated sperm essentially results from two major waves of epigenetic reprogramming, occurring after fertilisation and during germ cell differentiation [24, 25]. Both are incredibly sensitive to various internal and external factors that may modulate DNA methylation and affect the ability of sperm to establish a pregnancy [26, 27] as well as the health of the offspring [18, 28].

While there is no consensus about what level of global DNA methylation is beneficial for sperm or embryos, studies are focused on detailed investigation of DNA methylation and its association with various regions across the genome. Human studies have shown a relationship between DNA methylation patterns and sperm quality, represented by motility, morphology and DNA fragmentation [29–31]. Differential DNA methylation patterns were observed in sperm of men with idiopathic infertility and in men whose partners suffered recurrent pregnancy failures [26, 32, 33]. However, there are only a limited number of studies focusing on the relationship between bull fertility and DNA methylation. Kropp et al. [34] analysed the methylome of sperm from pools of low and high-fertility bulls by Methyl-CpG-binding domain sequencing and showed that DNA was hypomethylated in sperm from low-fertility bulls; moreover, they identified 98 differentially expressed genes in blastocysts derived from sperm of those bulls. Takeda et al. [35] used a human methylation microarray and reported a relationship between sire conception rate and DNA methylation with 147 differently methylated cytosines (DMCs) and 10 differently methylated regions (DMRs) between low- and high-fertility Japanese Black bulls. The relationship of bull sperm quality and DNA methylation was reported by Capra et al. [36], who showed that sperm with low and high motility differed in DNA methylation of genes involved in the maintenance of chromatin structure. To analyse the DNA methylome the aforementioned study used reduced representative bisulphite sequencing (RRBS), which target specific regions of the genome. The same method was applied in a recent study, which reported a link between bull sperm DNA fragmentation, low-fertility and DNA hypermethylation [37]. Gross et al. [38] utilised whole-genome bisulphite sequencing and identified 1765 DMCs in sperm from low- compared to high-fertility bulls and highlighted 10 genes which may serve as predictors of bull fertility. However, even though these studies suggest that DNA methylation patterns regulate sperm function and the establishment of pregnancy, the association of DNA methylation and bull fertility is not consistent and calls for additional investigations.

Hence, we established a robust AI bull fertility model with bulls of divergent fertility based on a minimum of 500 inseminations per bull and tested the hypothesis that sperm derived from sires of distinct field fertility exhibit different sperm DNA methylation patterns.

## Results

### Sequencing quality controls

Reduced representation bisulphite sequencing generated an average of 30.3 (± 1.9) million reads per sample (Table 1). Furthermore, we identified 34.0 (± 0.21)% uniquely mapped reads. Bisulphite conversion rate, monitored using the conversion rate of the unmethylated cytosines added in vitro during the end-repair step of library preparation, was 99.62 (± 0.05)%. There was no difference for any of the parameters related to the quality of RRBS library between low- and high-fertility bulls (*p* > 0.05; Table 1). The remainder of the analysis focused on the 57.2 (± 1.43)% CpGs that were covered by at least 10 uniquely mapped reads, named as CpGs10, whose average methylation was comparable between both groups.

**Table 1:** Library characterisation, mapping efficiency on the bovine genome (ARS-UCD1.2), coverage and average methylation in reduced representative bisulphite sequencing (RRBS) libraries

	Low-fertility bulls (*n* = 10)	High-fertility bulls (*n* = 10)
Sequence pairs analysed (10^6^)	28.5 ± 2.52	32.2 ± 2.93
All maps (%)	88.0 ± 0.51	88.0 ± 0.61
Unique maps (%)	34.0 ± 0.30	34.0 ± 0.32
Ambiguous maps (%)	54.0 ± 0.32	54.0 ± 0.48
Total number of CpGs analysed (10^6^)	3.3 ± 0.04	3.4 ± 0.05
Percentage of CpGs covered by ≥10 sequences (CpGs10)	55.9 ± 2.25	58.5 ± 1.78
Mean genomic coverage by CpGs	19.7 ± 1.67	21.6 ± 1.80
Average DNA methylation of CpGs10 (%)	46.9 ± 0.41	47.6 ± 0.29
Average DNA methylation of CpGs without filter (%)	50.0 ± 0.35	50.4 ± 0.20
Bisulphite conversion rate (%)	99.6 ± 0.06	99.6 ± 0.09

Values are presented as mean ± standard error. CpGs10 are CpGs covered by at least 10 uniquely mapped reads. There was no difference between fertility groups in any of the parameters investigated (t-test, *p* > 0.05)

### Relation between bulls fertility and overall sperm DNA methylation profiles

**Fig. 1:**
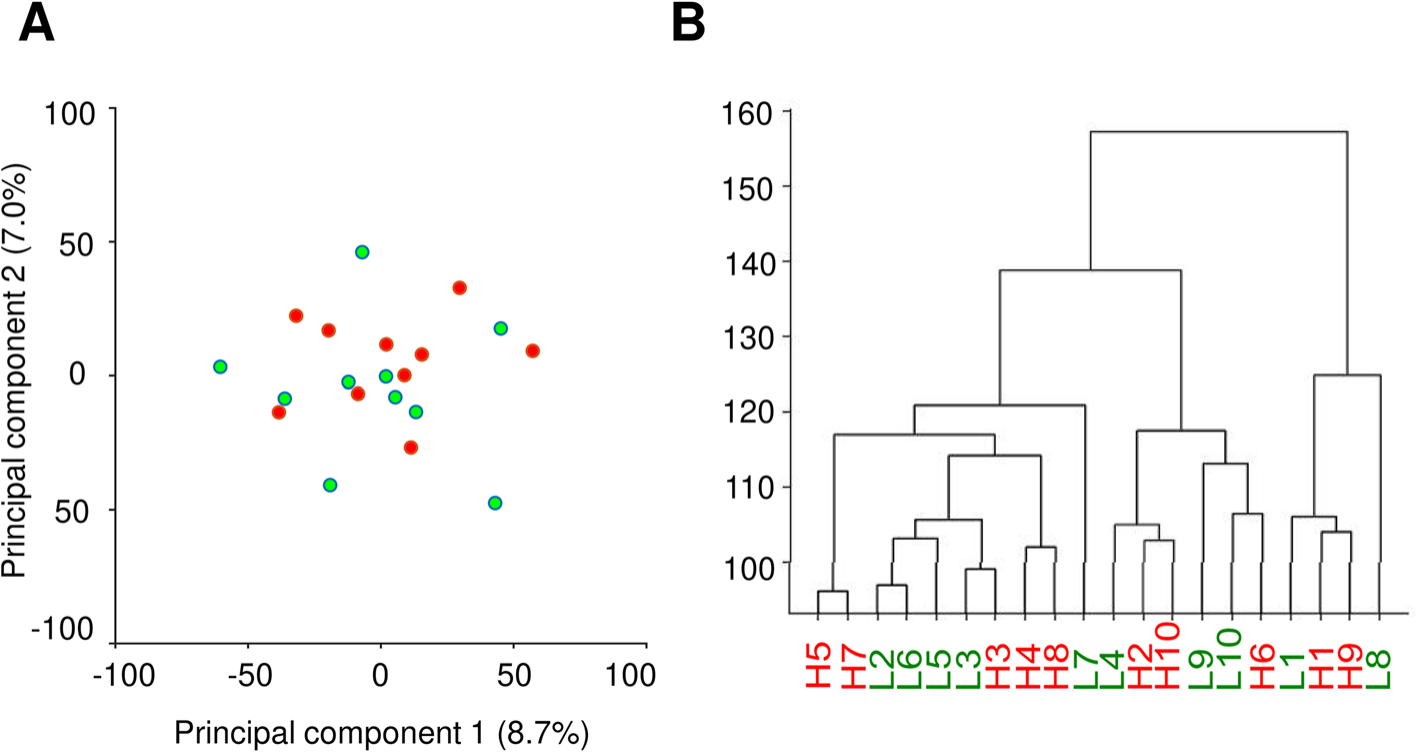
The relationship between bull fertility and sperm DNA methylation profile. (**A**) Principal component analysis (low-fertility bulls are displayed as green dots, high-fertility bulls as red dots) (**B**) Dendrogram clustering based on DNA methylation in sperm from all bulls (applied method: Ward method with Euclidean distance; L1 to L10, low-fertility bulls; H1 to H10, high-fertility bulls)

To assess the contribution of bull fertility to variations in the DNA methylation pattern, we performed principal component analysis (PCA) (Fig. 1A) and hierarchical clustering (Fig. 1B). Although we could distinguish three major clusters using a dendrogram (Fig. 1B), inter-individual variability in terms of DNA methylation was larger than intergroup resulting in no obvious clustering according to bull fertility status. To explore known effects of age on DNA methylation [39, 40], we analysed the PCA and hierarchical clustering results also with respect to age at ejaculate collection (Additional file 1: Fig. S1). These analyses demonstrate that in our dataset, neither fertility nor age of the bulls were major determinants of sperm DNA methylation patterns.

### Differentially methylated CpGs in the group of low- versus high-fertility bulls

**Fig. 2:**
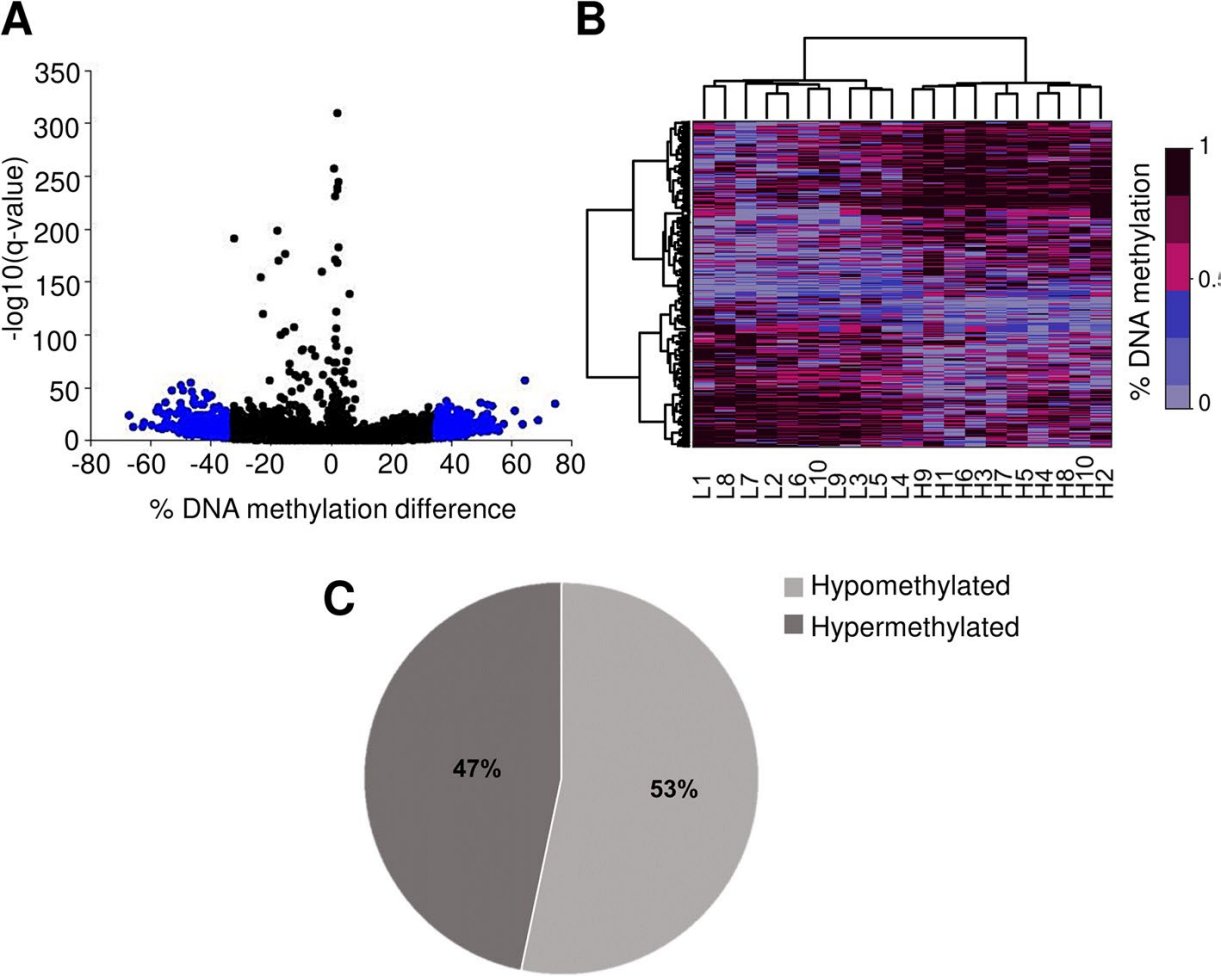
Differentially methylated cytosines (DMCs) in sperm from low- versus high-fertility bulls. (**A**) Volcano plot of DNA methylation difference between sperm from low- and high-fertility bulls. DMCs with DNA methylation difference > 35% and q-value < 0.001 (661 in total) are indicated in blue. (**B**) Heatmap clustering at the 661 DMCs. Rows correspond to individual DMCs and each column represents one bull (L1 to L10, low-fertility bulls; H1 to H10, high-fertility bulls). (**C**) Methylation status of DMCs in low-fertility bulls

Thereafter, differential analysis between fertility groups was conducted using methylKit with commonly used thresholds (DNA methylation difference ≥ 25% and q-value < 0.05) and we obtained 2805 DMCs and 72 DMRs (Additional file 2: Table S1). Spearman rank correlation test revealed a positive correlation (r = 0.53, *p* = 0.02) of average DNA methylation percentage at DMCs with bull age, suggesting that the age confounded the effect of fertility at these 2805 DMCs. Thus, we applied a stricter threshold. We filtered approximately 25% of the best-ranked DMCs and these were characterised by a DNA methylation difference ≥ 35% and a q-value < 0.001. These criteria were used for repeated differential analysis and resulted in the identification of 661 DMCs and 10 DMRs (Fig. 2A, Additional file 3: Table S2). The correlation between average DNA methylation percentage at DMCs with bull age was no longer significant (r = 0.33; *p* = 0.16), suggesting that the confounding effect of age was mostly eliminated. As the aim of this study was to examine the effect of bull fertility on the DNA methylation of sperm, DMCs, which were excluded after applying of more stringent criteria because of age correlation, were not considered further.

**Fig. 3:**
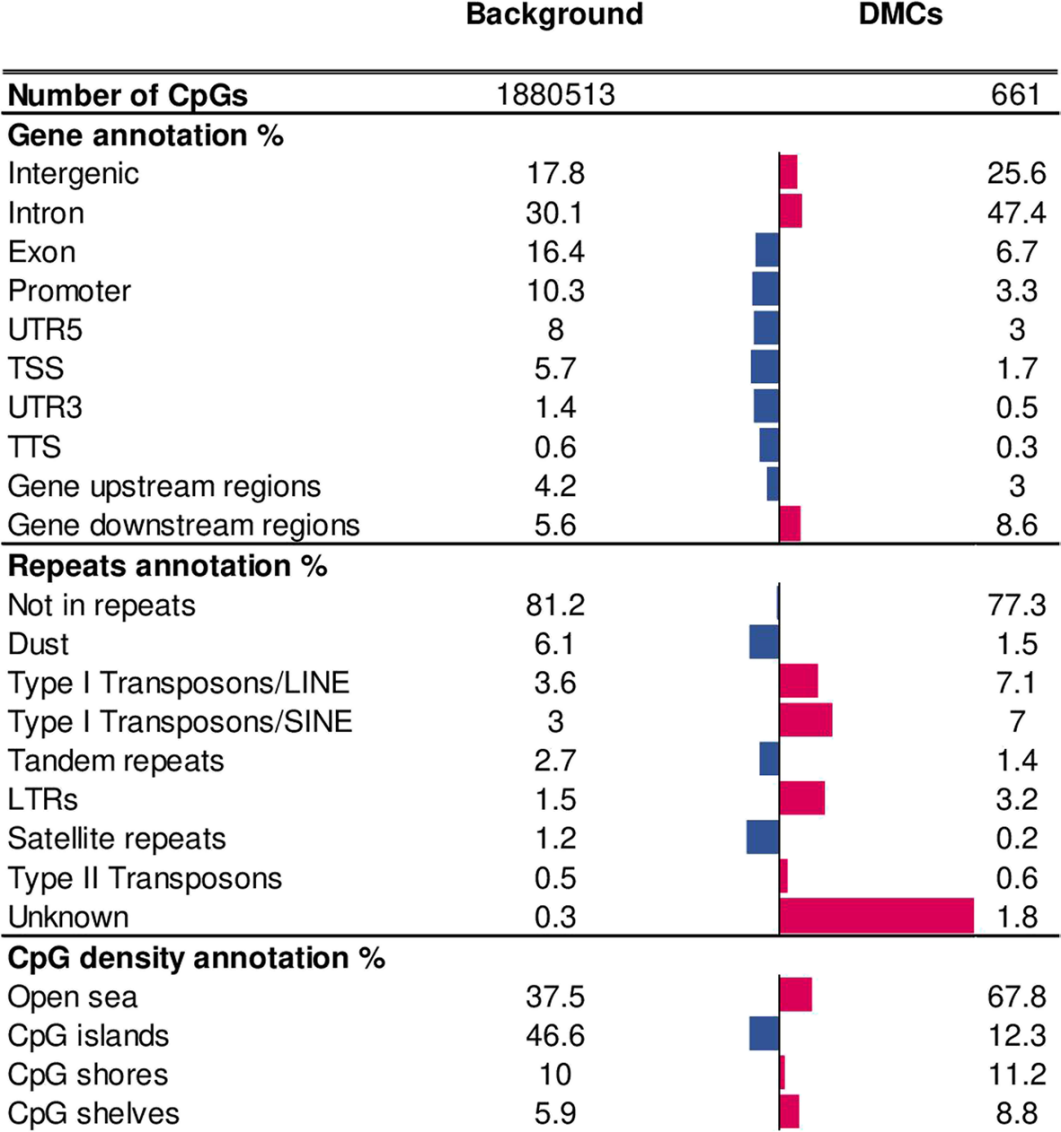
Enrichment of gene features, repetitive elements and CpG-rich regions within DMCs in sperm from low-versus high-fertility bulls compared to the background (all CpGs10 covered in at least five samples per group). Bar charts represent relative percent enrichment (pink) or depletion (blue) in DMCs compared to the background (5’untranslated regions (UTR5); 3’untranslated regions (UTR3); transcriptional termination site (TTS); transcriptional start site (TSS); long interspersed elements (LINEs); short interspersed nuclear elements (SINEs); long terminal repeat elements (LTRs))

**Fig. 4:**
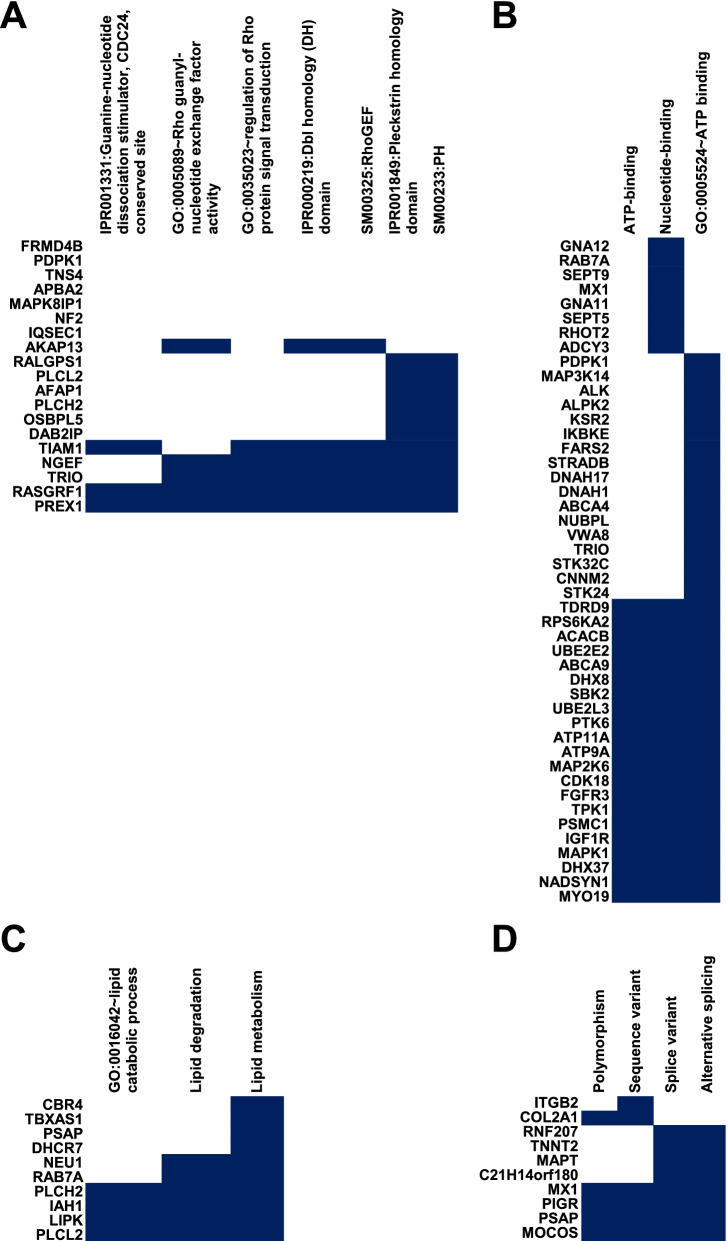
Enrichment analysis using the DAVID bioinformatics tool was focused on genes differentially methylated in sperm from low- versus high-fertility bulls. As a reference, the list of all genes covered by reduced representative bisulphite sequencing (19,962 genes) was used. (**A**) The first diagram (EASE score 2.07) represents genes clustering across categories related to Pleckstrin homology-like domain and Rho guanine nucleotide exchange factor (**B**) The second diagram (EASE score = 1.89) represents a cluster of genes in categories related to ATP and nucleotide binding activity. (**C**) The third diagram (EASE score = 1.85) represents a cluster of genes in categories related to lipid metabolism and degradation (**D**) The fourth diagram (EASE score = 1.78) represents a cluster of genes in categories polymorphisms and splicing. Default settings of the DAVID bioinformatics tool were applied and clusters of genes with EASE enrichment score > 1.3 were considered as significant. The blue colour illustrates that the listed gene occurred within each category

When the hierarchical clustering was performed on these 661 DMCs, the bulls were clearly segregated according to fertility (Fig. 2B). The ratio of hypomethylated (53%) and hypermethylated (47%) DMCs in low-fertility bulls was relatively balanced when compared to high-fertility bulls (Fig. 2C). All DMCs were annotated relative to genes and other genome features (Additional file 3: Table S2). We then investigated whether DMCs were enriched for specific genome features compared to the background, which included all the CpGs10 covered by RRBS in at least 5 bulls per group (Fig. 3). While the DMCs were depleted in most gene features with the exception of introns and gene downstream regions, repetitive elements, such as long interspersed elements (LINEs), short interspersed nuclear elements (SINEs), long terminal repeat elements (LTRs) and Type II Transposons were found to be enriched among DMCs. Regarding the regions distinguished based on the CpG density, CpG shelves, shores and open sea were overrepresented within DMCs when compared to the background. Gene ontology analysis using the DAVID bioinformatics tool was performed on the 363 genes containing at least one DMC and only the clusters with EASE enrichment score more than 1.3 [41] were considered as significant (Fig. 4). Based on these criteria, 78 unique genes with average methylation difference 41 ± 0.7% and q-value < 0.001 were classified into different categories related to “pleckstrin homology-like domain”, “Rho guanine nucleotide exchange factor”, “ATP and nucleotide binding”, “lipid metabolism” as well as “polymorphisms” and “splicing”.

### Fertility-related differentially methylated regions

**Fig. 5:**
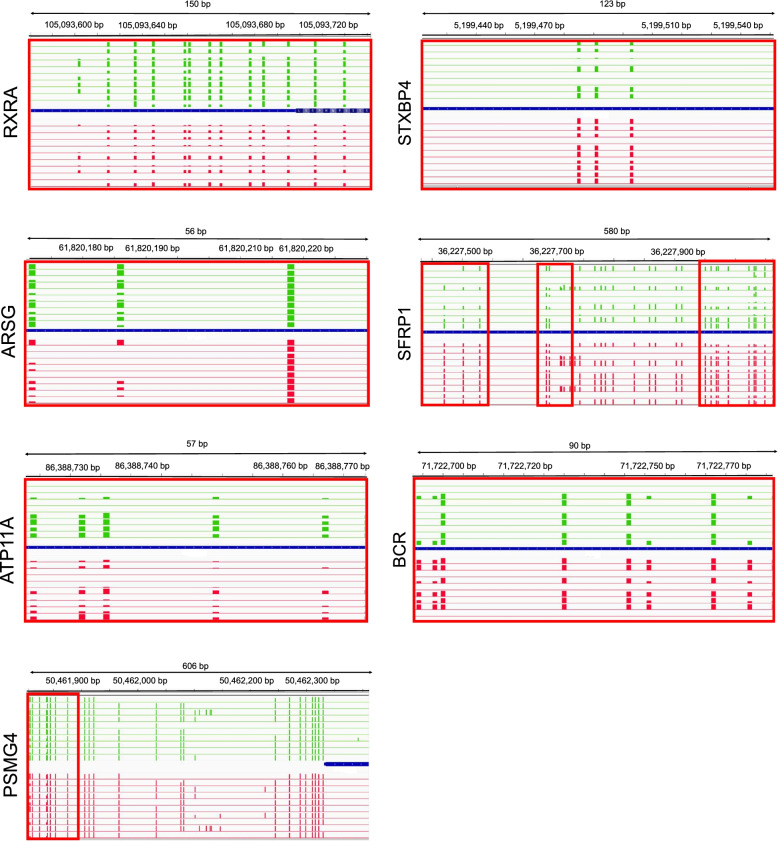
Graphically displayed differently methylated regions (DMRs) related to seven unique genes. These captures were performed by Integrative Genomic Viewer software. The bar charts represent the methylation percentages at each CpG10 position for high-fertility (red) and low-fertility (green) bulls. The position of the DMRs is highlighted by the red rectangles

Regarding identified 10 DMRs (Additional file 3: Table S2), there was a similar trend to the DMCs and the ratio of hypo- to hyper-methylation was quite balanced. Indeed 6 of the 10 DMRs were hypomethylated in low-fertility bulls when compared with high-fertility bulls. In relation to various annotated regions, 90% were overlapped with regions linked to genes, especially introns, and 60% were overlapped with repetitive elements (SINEs, LINEs, Tandem repeats). Based on CpG density, the same percentage of DMRs were mapped in open sea (40%) and islands (40%) with the remainder in shore and shelve regions. Within DMRs, 7 unique genes were identified (*SFRP1, ATP11A, ARSG, PSMG4, BCR, STXBP4, RXRA*) (Fig. 5). While *ATP11A, ARSG, PSMG4* and *RXRA* were hypermethylated, *BCR, STXBP4* and *SFRP1* were hypomethylated in the low-fertility bulls. Although, most of the DMRs occurred within introns of these genes, the DMR related to *RXRA* was localised in an exon and the DMR related to *PSMG4* in a downstream gene region.

## Discussion

Reprogramming of the gamete epigenome after fertilisation plays a central role in the acquisition of totipotency and in embryonic genome activation. A plethora of studies have described the relationship of the sperm epigenome to the success of pregnancy establishment. Completed protamination, post-translational modifications of histones as well as non-coding RNAs have undeniable impact on the progression of early embryo development [19–23]. Alterations to the sperm methylome may also impact these processes with consequences for pregnancy establishment and post-natal live [28, 42]. Methylation pattern has previously been related to sperm quality in various species and has been proposed to be a promising predictor of fertility [34, 43, 44]. However, the role of sperm DNA methylation in bull field fertility is poorly understood. This study focused on identifying differences in DNA methylation pattern using RRBS between low- and high-fertility bulls. Using stringent criteria, (DNA methylation > 35% and q-value < 0.001), we showed that sperm DNA methylation at certain CpGs is related to bull fertility. We identified 661 DMCs, 10 DMRs and highlighted seven differently methylated genes in sperm from low versus high-fertility bulls.

Despite this, hierarchical clustering did not reveal obvious discrimination based on fertility status with high levels of inter-individual variance. Similar trends were previously reported in boar and bull sperm [43, 45]. Within identified DMCs, hypomethylation and hypermethylation were represented almost equally (53 and 47%, respectively). This is in agreement with other studies that found quite a balanced ratio between hypomethylated and hypermethylated DMCs [34, 38, 45] and suggest that the level of methylation itself does not reflect fertility in cattle. In contrary, studies in human reported DNA hypermethylation in sperm of males with clinically proven infertility [46–48]. Thus, it must be noted that while the most of studies on sperm DNA methylation were done in infertile males, this study worked with subfertile bulls, but not infertile. This means that while the bulls were divergent in fertility, all bulls passed post-thaw sperm quality assessments.

Regarding the distribution of detected DMCs across the genome, 25.6% were intergenic and 67.8% occurred in open sea. This is in agreement with other studies in bulls, boars and humans [43, 48, 49]. In contrary, Narud et al. [37] found the majority of DMCs in intergenic regions. This could potentially be explained by different breeds with Norwegian Red bulls being used in this study compared with Holstein Friesians used in the current study. Indeed, animal breeds effect on DNA methylation was recently found in pigs [50, 51]. Furthermore, repetitive elements, such as SINEs, LINEs and LTRs were over-represented within DMCs. Distinct methylation of repetitive elements was observed in infertile males [52]. Interestingly LINE1 and SINEs preserve their nucleosome structure within sperm chromatin and because it is known that nucleosome distribution in sperm genome is not random [53, 54], their involvement in embryo development is likely [55], especially when we consider that some of them escape demethylation in early embryos [24, 56]. More recently, SINEs and LINEs were found to contribute in the transition in embryos from the 2-cell to the 4 cell stage as well as in embryonic genome activation [57, 58].

Enrichment analysis for DMCs was performed using the DAVID bioinformatics tool. We identified four gene categories covering 78 genes, namely “pleckstrin homology-like domain” and “Rho guanine nucleotide exchange factor”, “ATP and nucleotide binding”, “lipid metabolisms”, “polymorphisms” and “splicing”. Genes involved in categories “pleckstrin homology-like domain” and “Rho guanine nucleotide exchange factor”, are involved in the organisation of cytoskeleton and intracellular signalling [59, 60], which is especially important during early embryo development [61, 62]. In contrast, genes involved in “ATP and nucleotide binding” are more general and represent those encoding proteins that are dependent on ATP synthesis. Within this category, there are genes coding for sperm cytoskeletal proteins (*DNAH1, DNAH17*) [63–65] but also various enzymes that are indispensable for spermatogenesis and embryo development (*IGF1R, UBE2E2, MAPK1*) [66–69]. Genes involved in “lipid metabolism” code proteins that are likely to be involved in capacitation and embryonic development [70, 71].

We identified 10 DMRs, which correspond with seven genes (*SFRP1, RXRA, ATP11A, STXBP4, BCR, ARSG, PSMG4*) that may contribute to the variation in bull fertility. While *ATP11A, ARSG, RXRA* and *PSMG4* were found to be hypermethylated, *BCR, SFRP1* and *SRXBP4* were hypomethylated in sperm from low-fertility bulls. Glycoprotein *SFRP1* modulates the Wnt signalling pathway, which is involved in spermatogenesis and epididymal sperm maturation, but also embryonic sexual development [72–75]. In mice, *SFRP1* regulates spermatid adhesion as well as their release during spermiation in the testes [76]. Moreover, this protein was also detected in embryonic testes and a mouse knock-out model showed malformation in development of the testes and impaired maturation of the reproductive tract [75]. The expression of *SFRP1* was also found in the trophoblast, and through Wnt signalling it is possibly involved in placental development [77]. This is further supported by Partl et al. [78] who detected overexpression of *SFRP1* in abnormal human placentas compared to healthy controls. During spermatogenesis, embryonic development and placentation, *RXRA* acts as a transcription factor. Through interaction with retinoic acid receptors, *RXRA* regulates various biological functions, such as cell development, differentiation and apoptosis [79, 80]. *RXRA* was detected in Sertoli cells and germ cells within the testes, with under-expression found in infertile men [81]. Similarly, depletion of *RXRA* leads to infertility in male mice [82] indicating a crucial role of *RXRA* for healthy sperm development. *RXRA* regulates embryonal development too [79, 83] and mRNA of *RXRA* was detected at all stages of bovine embryos in both the inner cell mass and trophectoderm [84].

Similarly, *BCR* and *STXBP4* are expressed in bovine 8-cell embryos and blastocysts [85]. Even though the role of these genes is unknown in the context of sperm influence on embryonic development, they are involved in basic cell functions. While *BCR* contributes to cell division and migration [62], *STXBP4* regulates glucose metabolism through binding to syntaxin 4 [86] and is a negative regulator of the Hippo signalling pathway that is involved in cell proliferation and apoptosis [87, 88]. Therefore, a role during spermatogenesis or early embryonic development is likely.

The gene *ATP11A*, which was hypermethylated in sperm from low-fertility bulls, encodes a protein that is an integral part of the cell membrane and facilitates translocation of phosphatidylserine from the outer to the inner layer of the membrane. Accordingly, it is responsible for membrane stability, cholesterol homeostasis, cell proliferation and apoptosis [89, 90]. Expression of this gene was detected in mouse testes [90] and 16-cell bovine embryos [85]. Embryos generated from conditional knockout mice of *ATP11A* have abnormalities in neurological development and delays in development. A similar impact on impaired embryonic development has pointed to a mutation of this gene in humans [91, 92].

*ARSG*, also hypermethylated in sperm from low-fertility bulls, codes for a sulfatase that is responsible for the degradation of 3-O-sulfated N-sulfoglucosamine residues of heparan sulfate glycosaminoglycans [93, 94], which occurs in the endometrium and regulates signalling, leading to receptiveness of the uterus to the blastocyst [95]. *PSMG4* codes for one of chaperones responsible for proteasome assembly [96]. Proteasome as part of ubiquitin-proteasome system is involved in degradation and recycling of proteins during spermatogenesis, epididymal maturation, capacitation, but also during fertilisation and embryonic development [97–100]. Therefore, an aberration of *PSMG4* expression may lead to defects in the ubiquitin-proteasome system and result in subfertility.

A mutual feature of all these genes is that their role is most likely during early embryonic development. Although the paternal methylome is substantially reprogrammed after fertilisation, specific CpGs preserve their methylation pattern from sperm to the blastocyst [101, 102]. Hence, it is reasonable to hypothesise that the altered methylation we observed in sperm of subfertile bulls leads to differences in the expression of these genes, with potential impacts on early development. To test this, it would be interesting to track these genes and their expression in embryos to demonstrate the effect of the differential methylation pattern in sperm on embryonic development.

## Conclusion

To conclude, results of the current study demonstrate that sperm DNA methylation at certain CpGs is related to bull fertility and seven differently methylated genes occurred within DMRs have been reported to regulate spermatogenesis and embryonic development and thus may contribute to varying bull fertility.

## Methods

### Animals and semen collection

Mature Holstein-Friesian bulls with high (*n* = 10) or low (*n* = 10) fertility were selected from a population of 1665 AI bulls (Additional file 4: Table S3). Bull fertility was assessed based on adjusted fertility scores [103], calculated based on calving rates by the Irish Cattle Breeding Federation from a record of at least 500 inseminations (mean = 13,292, min = 519, max = 100,288). The sire fertility model used in Ireland is an animal adjusted model [103], typical of the model used in many other countries. This is a multiple regression mixed model that considers numerous fixed effects such as number of inseminations, year and month of service, days since calving and cow parity, non-additive effects such as heterosis, semen type (fresh or frozen) and random effects such as cow breed, cow genotype, AI technician, herd and service bull. High-fertility bulls had an average adjusted fertility score of + 6.5%, whereas low-fertility bulls had an average fertility score of − 6.6%. The mean of the population was zero. Semen was collected via an artificial vagina, frozen in 0.25-ml French straws using a programmable freezer and stored in liquid nitrogen pending further analysis.

### DNA isolation

Three frozen semen straws representing three ejaculates (1 straw per ejaculate) per bull were used for DNA extraction. After thawing at 37 °C, the thawed semen was first washed with phosphate-buffered saline (PBS) to remove the semen extender and then with deionised H_2_O to eliminate somatic cells. Prior to lysis, the absence of somatic cells was confirmed by visualising a sample under a phase contrast microscope. Subsequently, the sperm pellet was resuspended in 200 μl lysis buffer [49] in the presence of 0.2 mg/ml proteinase K, and incubated overnight at 55 °C. After incubation with 25 μg/ml RNAse A for 1 h at 37 °C, genomic DNA was extracted twice using phenol and phenol:chloroform (1:1), then ethanol-precipitated and washed. The dried pellet was resuspended in TE buffer (10 mM Tris HCl pH 7.5, 2 mM EDTA) and the DNA concentration was measured using a Qubit 2.0 Fluorometer with the dsDNA BR Assay kit (Invitrogen, Renfrew, UK).

### Reduced representative bisulphite sequencing

RRBS libraries from the 10 low- and 10 high-fertility bulls were prepared from 200 ng of genomic DNA digested with MspI (Fermentas, Schlangen, Germany) using a semi-automated procedure [49]. Briefly, after ligation to 55 bp methylated Illumina adapters for paired-end sequencing, deionised H_2_O was added up to 50 μl, which was followed by size selection using SPRI select magnetic beads (Beckman Coulter) in order to obtain fragments ranging from 150 to 400 bp (40–290 bp genomic DNA fragments + adapters). The DNA was then converted twice with sodium bisulphite using the EpiTect bisulphite kit (Qiagen, Manchester, UK) following the manufacturer’s instructions. Converted DNA was amplified with Pfu Turbo Cx hotstart DNA polymerase (Agilent, CA, US) using 14 PCR cycles. The libraries were finally purified using AMPure XP beads (Beckman-Coulter, Maryfort, Ireland) and sequenced on an Illumina Novaseq6000 sequencer to produce 100 bp paired-end reads (Integragen SA, Évry, France).

### Bioinformatic analysis

The sequences displayed the expected nucleotide composition based on MspI digestion and bisulphite conversion according to FastQC quality control. Subsequent quality checks and trimming were carried out using Trim Galore v0.4.5, which removed adapter sequences, poor quality bases and reads (Phred score below 20) and reads shorter than 20 nucleotides. High quality reads were aligned to the bovine reference genome (ARS-UCD1.2) in which the sequence of the Y chromosome has been incorporated (GenBank: CM011803.1) using Bismark v0.20.0 in the default mode with Bowtie 1 [104, 105]. The sequence of Y chromosome was extracted from the paternal haplotype from a *Bos taurus* x *Bos indicus* hybrid (UOA_Angus_1 assembly). The bisulphite conversion rate was estimated from the unmethylated cytosine added in vitro during the end-repair step and was on average 99.6%. The CpGs were then selected based on their coverage by uniquely mapped reads. Only CpGs covered by at least 10 uniquely mapped reads (termed as CpGs10) were retained for subsequent analyses. Each CpG10 was assigned a methylation percentage per sample calculated from Bismark methylation calling: [(reads with “C”)/(reads with “C” + reads with “T”)] × 100, which could be visualised using the Integrative Genome Viewer (IGV) genome browser [106]. The groups of low- and high-fertility bulls were compared for the mapping efficiency, coverage, and average methylation at CpGs10 using t-test. For Fig. 1, euclidean hierarchical clustering was computed on the matrix of methylation percentages for each CpG10 covered in at least five bulls per group (background, which represents 1,880,513 CpGs10). For each comparison, DMCs were identified from the background using methylKit v1.0.0 software in the default mode [107]. Initially the threshold was set at an adjusted *p*-value (q-value) less than 0.05, and the methylation difference between two groups of at least 25% (Additional file 2: Table S1). However, because of the significant correlation of bull age and DNA methylation difference, more stringent criteria were then applied. Final analysis was done with the q-value less than 0.001 and the methylation difference at least 35% (Additional file 3: Table S2). A DMR was constituted by a minimum of three DMCs with a maximum inter-DMC distance of 100 bp.

The annotation of the DMCs, DMRs, and the 1,880,513 background CpGs10 was performed as described by Perrier et al .[49] relative to gene features, CpG density and repetitive elements using an in-house pipeline. The reference files were uploaded from Ensembl (ftp://ftp.ensembl.org/pub, release 102). The following criteria were applied: TSS, − 100 to + 100 bp relative to the transcription start site (TSS); promoter, − 2000 to − 100 bp relative to the TSS; TTS: − 100 to + 100 bp relative to the transcription termination site (TTS); upstream gene region, − 10 to − 2 kb from the TSS; downstream gene regions, + 100 bp to + 10 kb from the TTS; shore, up to 2000 bp from a CpG island (CGI); and shelf, up to 2000 bp from a shore. A site/fragment was considered to belong to a CGI (respective shore and shelf) if an overlap of at least 75% was observed between the site/fragment and the CGI (respective shore and shelf). A site/fragment was considered as being overlapped by a repetitive element whatever the extent of this overlapping. The list of annotated DMCs and DMRs is available in Additional file 2: Table S1 and Additional file 3: Table S2. Genes containing DMCs at a maximal distance of 10 kb were subjected to enrichment analyses with the Database for Annotation, Visualization, and Integrated Discovery (DAVID) using default parameters [41], and using the gene list covered by the 1,880,513 background CpGs10 as the reference. Clusters of terms showing EASE enrichment scores above 1.3 were considered significant. All other statistical analysis related to graphs plotting were computing using MATLAB software v.R2021a (The MathWorks Inc., Natick, MA, USA).

## Supplementary Information

**Additional file 1.**

**Additional file 2.**

**Additional file 3.**

**Additional file 4.**

## Data Availability

Additional data can be found in supplementary files. RRBS fastq files have been deposited in the European Nucleotide Archive (ENA) at EMBL-EBI under accession number PRJEB49406 (https://www.ebi.ac.uk/ena/data/view/ PRJEB49406).
