# Supplementary material for: Sperm DNA methylation patterns at discrete CpGs and genes involved in embryonic development are related to bull fertility
Source: BMC Genomics. 2022 May 18;23:379. doi: 10.1186/s12864-022-08614-5 (PMC9118845; doi:10.1186/s12864-022-08614-5)
Supplement: Supplementary file 1 — Additional file 1. [file 12864_2022_8614_MOESM1_ESM.pdf]

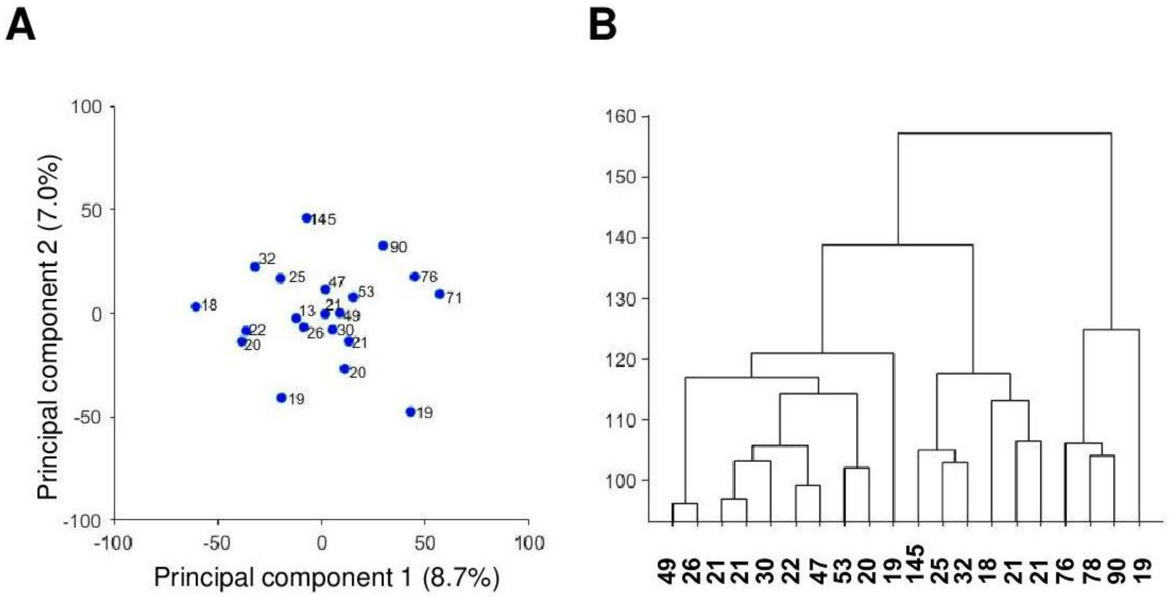

Additional file 1: Figure S1. Unsupervised clustering of DNA methylation in sperm according to age of individual bulls (A) Principal component analysis (B) Dendrogram clustering based on DNA methylation in sperm from all bulls (applied method: Ward method with Euclidean distance).
